# Parental Adverse Childhood Experiences and Health Care Use Among Children With Sickle Cell Disease

**DOI:** 10.1001/jamanetworkopen.2025.19793

**Published:** 2025-07-10

**Authors:** David K. Wilson, Shelley Crary, Gabbey Hobbs, Trenesha L. Hill, Beverly Spray, Rachel Mayo, Andrew Tran, Joana Mack, Divyaswathi Citla-Sridhar, Suzanne Saccente, Ellen van der Plas

**Affiliations:** 1 Department of Pediatrics, University of Arkansas for Medical Sciences, Little Rock; 2 Division of Hematology/Oncology, Arkansas Children’s Hospital, Little Rock; 3 Arkansas Children’s Research Institute, Little Rock

## Abstract

**Importance:**

Adverse childhood experiences (ACEs) are associated with poor health care use. In African American communities, where ACEs are more prevalent, it is critical to understand the association of ACEs with chronic illnesses, such as sickle cell disease (SCD), in children to expand trauma-informed care and improve outcomes.

**Objective:**

To examine the association between parental ACEs and health care use in pediatric patients with SCD, accounting for factors that may influence this association.

**Design, Setting, and Participants:**

This unblinded cross-sectional study was conducted from January 1, 2020, to December 31, 2023, at Arkansas Children’s Hospital. Seventy-two of 77 approached caregivers (93.5%) participated. All resided within the Arkansas Children’s Hospital catchment area.

**Exposure:**

Parental ACEs and resiliency were assessed using the Adverse Childhood Experiences Questionnaire (ACE-Q) and the Brief Resiliency Scale, respectively. Neighborhood deprivation was assessed using the Area Deprivation Index (ADI).

**Main Outcomes and Measures:**

Primary outcomes included number of visits to the emergency department (ED), number of missed clinic visits, and medication adherence (categorical outcomes). Independent variables included parental ACEs, parental resilience, and neighborhood ADI. Coefficients were derived from mixed regression models.

**Results:**

The final sample included 79 observations (mean [SD] patient age, 9.73 [4.88] years; 44 [55.7%] female) from 72 caregivers (6 reporting for >1 child), with 70 (88.6%) of ACE-Q respondents being mothers. The ACE risk was low in 32 caregivers (40.5%), intermediate in 33 (41.8%), and high in 14 (17.7%). Patients whose caregivers were at high risk had significantly more ED visits and admissions compared with those whose caregivers were at low risk (regression coefficient = 7.35; 95% CI, 1.77-12.94). Likewise, the number of ED visits and admissions was higher among patients whose caregivers had lower resiliency compared with those with more resilient caregivers (regression coefficient = 5.69; 95% CI, 0.13-11.26). ADI was not significantly associated with ED visits and admissions (regression coefficient = −0.02; 95% CI, −0.12 to 0.08). Parental ACEs were not significantly associated with other metrics of health care use.

**Conclusions and Relevance:**

This cross-sectional study of ACEs and resiliency in parents of children with SCD found that higher levels of parental ACE scores and lower resiliency were associated with visits to the ED. These findings support the importance of screening caregivers for ACEs and resilience to enhance trauma-informed care, particularly in marginalized populations.

## Key Points

**Question:**

Are parents’ adverse childhood experiences (ACEs) associated with health care use for their children with sickle cell disease?

**Findings:**

In this cross-sectional study of 79 patients with sickle cell disease and their primary caregivers, more parental ACEs and lower resiliency were associated with increased number of visits to the emergency department.

**Meaning:**

The association between parental ACEs and hospital visits underscore the need for trauma-informed approaches to improve health care delivery.

## Teaser

This cross-sectional study examines the association between parental adverse childhood experiences and health care use in pediatric patients with sickle cell disease.

## Introduction

The landmark adverse childhood experience (ACE) study from Kaiser Permanente in 1998 established a relationship between chronic diseases in adulthood and exposure to emotional, physical, or sexual abuse in childhood.^1^ In addition to increasing the risk of developing chronic illness,^2,3^ ACEs also affect health care use.^3,4^ Adults and children with any reported ACEs are more likely to not go to appointments or use emergency services than those with no ACEs.^5,6^ These findings have led to an increasing awareness among health care professionals for monitoring patients’ ACEs to ensure effective health care delivery and use,^7^ particularly in at-risk populations.

Sickle cell disease (SCD) is the most common inherited blood disorder among individuals of African ancestry.^8^ The gene mutation in SCD causes red blood cells to become sickle-shaped, which can block blood flow and reduce oxygen delivery. As a result, patients experience numerous debilitating and life-threatening complications throughout their lifetime, including chronic hemolytic anemia, unpredictable episodes of pain, and widespread organ damage.^9,10,11,12,13^ Prevention of pain episodes and other complications of SCD (eg, high risk of stroke)^12,14^ requires comprehensive care from a hematologist. However, patients with SCD often do not receive the care they need.^15,16,17,18^ Given the profound health burden of SCD on patients and families, eliminating barriers to effective care is essential. Achieving this goal likely requires a comprehensive approach that not only accounts for clinical needs but also recognizes the social and psychological influences on health—particularly ACEs, which disproportionally affect African American communities.^1,19^ Within the context of SCD, psychological stress increases health care use among patients with SCD,^20^ and ACEs have been associated with increased morbidity.^17,21^ Addressing the role of ACEs in health care use in SCD may contribute to ensuring optimal care for patients.

Evidence of the importance of recognizing ACEs in patients continues to mount,^22,23,24^ although knowledge on the potential impact of parental ACEs on health care use in a pediatric setting is limited. Providing effective care for pediatric patients necessitates an understanding of the experiences of both the patient and their caregivers.^25^ For example, Eismann and colleagues^26^ found that maternal ACE exposure was associated with missed well-child visits by the time the child reached 2 years of age.^26^ Likewise, children of caregivers with 4 or more ACEs had significantly higher odds of unanticipated health care reuse relative to children of caregivers with no ACEs.^27^

The broader context in which ACEs occur should also be considered, particularly the role of socioeconomic adversity and the potential protective effects of resilience. Although ACEs occur at any socioeconomic level, higher ACE scores and economic adversity are associated with each other.^28,29^ Material hardship, such as housing instability or lack of transportation, has also been identified as an important risk factor in poor health care use.^30,31^ However, resilience factors, including family resilience and neighborhood cohesion, may help buffer these effects and promote more consistent engagement with health care systems despite adversity.^30^ Integrating an understanding of both risk and resilience into pediatric care may be essential for improving outcomes among families affected by SCD and ACEs. Thus, the aim of the current study was to characterize associations between parental ACEs and health care use in pediatric SCD, while simultaneously considering pertinent factors that are associated with health care use, including socioeconomic adversity and parental resiliency. We hypothesized that parental ACEs would adversely affect health care use in pediatric patients with SCD.

## Methods

### Population

The population of this cross-sectional study consisted of primary caregivers and their children with SCD receiving care at the Arkansas Children’s Hospital (ACH) sickle cell outpatient clinic. Caregivers provided survey responses, whereas demographic information and health care use metrics pertaining to the children were recorded from electronic medical records. To be eligible, caregivers had to be the legal guardian of a child 17 years or younger with a confirmed SCD diagnosis. Caregivers with multiple children who met these criteria were eligible (eTable 1 in Supplement 1). Caregivers were approached during a clinic visit from January 1, 2020, to December 31, 2023, and invited to complete the Adverse Childhood Experiences Questionnaire (ACE-Q) and Brief Resilience Scale. Investigators approached 77 caregivers, 72 of whom agreed to participate (93.5%). This cross-sectional study adhered to the Strengthening the Reporting of Observational Studies in Epidemiology (STROBE) reporting guideline.

Study procedures were explained to caregivers while they attended their child’s clinic visit. Caregivers provided verbal consent prior to completing the study procedures and were not incentivized for participating. Written informed consent was waived to limit potential linkage between the identity of the respondents and the sensitive data contained in the ACE-Q. The study was approved by the institutional review board of the University of Arkansas for Medical Sciences.

### Dependent Variables

Retrospective medical record review was conducted manually on each patient for records collected up until the date of enrollment in this study (2002-2023), including number of missed visits, visits to the emergency department (ED), ED admissions, therapy refusal (obtained from clinic notes), and number of transfusions. ED visits and admissions were combined into a single variable to limit the number of models being conducted. Therapy refusal was defined as a caregiver or patient who ever refused a therapy recommended by the medical team. Adherence to hydroxyurea and penicillin therapy was also abstracted from pharmacy refill data. Patients who filled their prescription 75% or less of the time were considered not adherent, whereas those filled their prescription more than 75% of the time were considered adherent.

### Independent Variables

#### Demographic Variables

Patients’ age, sex, and race and ethnicity were abstracted from the medical records. Data on race and ethnicity were collected because these data are relevant in the context of the disease being studied. The patients’ diagnosis was also obtained from medical records and included hemoglobin SS (n = 48), hemoglobin Sβ^0^ (n = 3), hemoglobin Sβ^+^ (n = 4), hemoglobin SC (n = 23), and hemoglobin SE (n = 1). We combined the former 2 categories into a single category called *severe genotype* and the latter 2 categories into *mild genotype*.

#### Adverse Childhood Events

Caregivers completed the ACE-Q, which includes 10 items pertaining to various types of trauma experienced before 18 years of age.^1^ The test-retest reliability for ACE scores was reported to be in the good to excellent range.^32,33,34^ Total scores on the ACE-Q range from 0 to 10 and were subsequently categorized using scoring guides into low risk (score of 0), intermediate (scores of 1-3), and high risk (score ≥4) bins to facilitate interpretation of the results in a clinically meaningful way.^2,29^

#### Brief Resilience Scale

The Brief Resilience Scale, which has demonstrated good internal consistency and test-retest reliability, was completed by caregivers and was developed to quantify the respondent’s ability to recover from stress.^35,36^ Scores range from 1 through 5 and are categorized per scoring interpretation guidelines as low resiliency (scores of 1-2.99), normal resiliency (scores of 3-4.30), and high resiliency (scores of 4.31-5).^36,37^ Given the relatively low number of caregivers in the high category (n = 17) and to facilitate interpretation of the coefficients, the normal and high categories were combined. The dichotomized variable included 2 levels: normal resiliency (scores of 3-5) and low resiliency (scores of 1-2.99).

#### Area Deprivation Index

The Area Deprivation Index (ADI) is created from publicly available data and provides a validated metric that ranks neighborhoods based on income, educational level, employment, and housing quality and accounts for age-, sex-, and race-specific factors in mortality.^22^ The ADI was initially developed in the late 1980s by the Health Resources and Services Administration to quantify families’ risk for adversity based on where they live. This information has been further refined and validated to 12-digit Federal Information Processing Series (FIPS) codes by the Center for Health Disparities Research in Wisconsin. The number of digits in the FIPS codes varies by level: state-level codes have 2 digits, whereas block group–level codes have 12, enabling precise identification of geographic areas within states and providing more granular data for socioeconomic analysis.^23^ These data are accessible through the University of Wisconsin Neighborhood Atlas via an online interactive tool. We used the downloadable dataset for the 2022 national ADI data (version 4) and linked patient addresses and FIPS codes to determine the ADI for each patient.^24^ The 2022 national ADI was derived from the 2018-2022 US Census American Community Survey Data and the 2022 Census Block Groups. National percentile rank scores range from 0 to 100, with higher scores representing higher levels of deprivation.

### Statistical Analysis

First, we conducted simple regression analyses for each dependent variable (eg, number of missed visits and number of ED visits) and ACE category (low vs intermediate and low vs high) as the independent variable. A threshold of *P* < .10 was used for this initial step to identify outcomes for multivariable analyses. Second, mixed multivariable analyses were performed on dependent variables that were significantly associated with ACE categories at the univariate level. Depending on the outcome, we ran linear (eg, number of ED visit) or logistic (eg, adherence) regression models. Mixed models included a random effect of caregiver to account for caregivers who had multiple children. Models were additionally adjusted for ADI and parental resiliency (normal resiliency vs lower resiliency). We explored the potential moderating effect of parental resiliency on health care use by adding an interaction term for parental ACEs and resiliency. Associations were considered significant at a 2-sided *P* < .05. Analyses were performed in R, version 4.2.1 (R Project for Statistical Computing).^38^

## Results

### Sample

**Figure 1.**
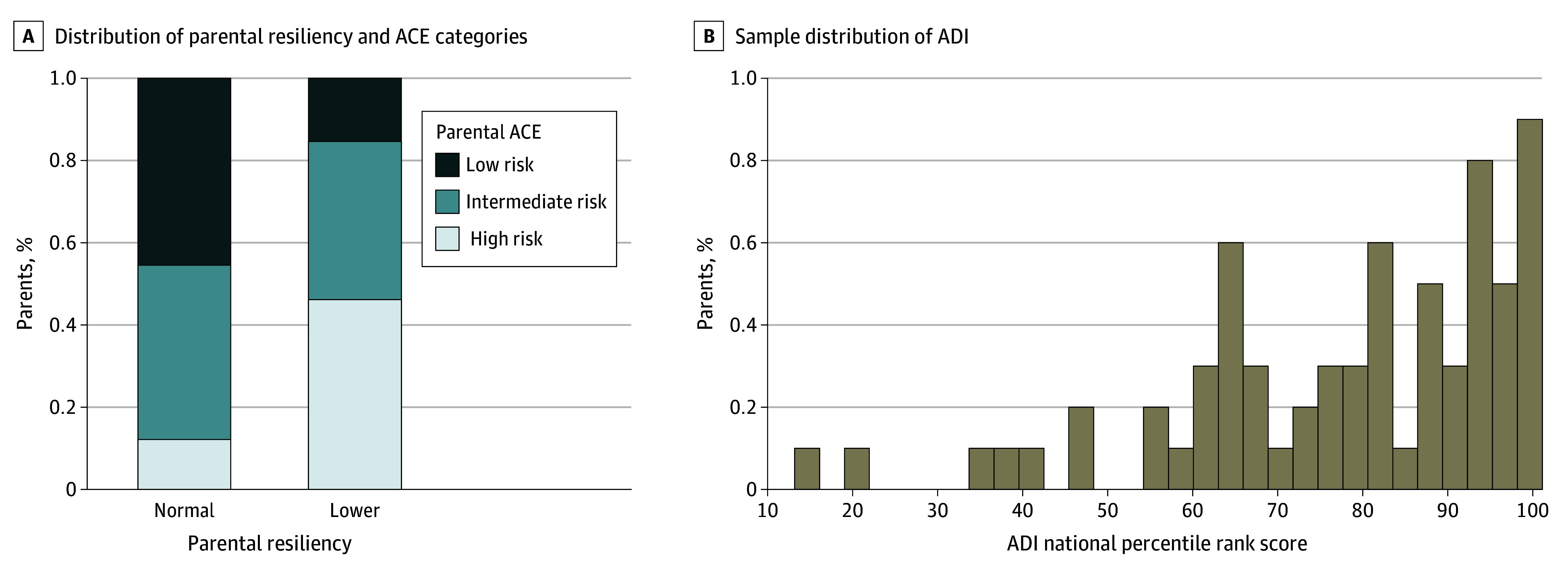
Distribution of Parental Resiliency and Adverse Childhood Experience (ACE) Categories and Area Deprivation Index (ADI) Percentile

The sample included 79 observations from 72 caregivers. Seventy (88.6%) of the ACE-Q respondents were mothers, a proportion that significantly deviated from the expected distribution (χ^2^_1_ = 47.1, *P* < .001). Patient age was a mean (SD) of 9.73 (4.88) years, and 48 (60.8%) had hemoglobin sickle cell disease. Forty-four patients (55.7%) were female and 35 were male (44.3%). Six caregivers provided data for 2 children or more (eTable 1 in Supplement 1). Table 1 summarizes key characteristics of the sample across parental ACE categories. In total, 32 caregivers (40.5%) were in the low-risk ACE category, 33 (41.8%) were at intermediate risk, and 14 (17.7%) were in the high-risk category (χ^2^_1_ = 8.68, *P* = .01). Sixty-six caregivers (83.5%) considered themselves to be resilient. Six caregivers (42.8%) in the high-risk ACE category had lower resiliency compared with 2 caregivers (6.3%) in the low-risk ACE category (χ^2^_1_ = 6.72, *P* = .01) (Figure 1A).

**Table 1.** Sample Characteristics Across Parental ACE Categories

Characteristic	Parental ACE category, No. (%)			*P* value
	Low risk (n = 32)	Intermediate risk (n = 33)	High risk (n = 14)	
Completed ACE-Q				
Mother	27 (84.4)	30 (90.9)	13 (92.9)	<.001
Other^a^	5 (15.6)	3 (9.1)	1 (7.1)	
Parental resiliency category				
Normal	30 (93.8)	28 (84.8)	8 (57.1)	.01
Lower	2 (6.3)	5 (15.2)	6 (42.9)	
Patient age, y				
Mean (SD)	8.43 (4.57)	11.2 (4.67)	9.21 (5.42)	.02
Median (range)	7.50 (1.92-17.0)	13.0 (0.750-18.0)	9.50 (2.00-19.0)	NA
Patient sex				
Female	20 (62.5)	18 (54.5)	6 (42.9)	.46
Male	12 (37.5)	15 (45.5)	8 (57.1)	
Patient ADI percentile				
Mean (SD)	73.5 (25.9)	81.2 (15.2)	79.5 (15.4)	.35
Median (range)	77.5 (15.0-100)	82.0 (48.0-100)	87.0 (48.0-99.0)	NA
Missing	6 (18.8)	4 (12.1)	1 (7.1)	NA
Patient SCD genotype				
Mild	11 (34.4)	12 (36.4)	5 (35.7)	.99
Severe	21 (65.6)	21 (63.6)	9 (64.3)	

^a^ Other category included fathers (7 [77.8%]) and grandparents (2 [22.2%]).

The mean (SD) ADI percentile across the entire sample was 77.96 (20.05) (Figure 1B). Parental ACE category was not significantly associated with ADI percentile (regression coefficient low vs intermediate risk = 7.67; 95% CI, −3.14 to 18.47; regression coefficient low vs high risk = 6.00; 95% CI, −7.59 to 19.59).

### Associations Between Health Care Use and Parental ACEs

Table 2 presents descriptive statistics and model parameters for parental ACE bins across outcome measures. On the basis of these results, the number of ED visits and admissions and number of missed clinic visits were analyzed using mixed multivariable models.^39,40^

**Table 2.** Descriptive Statistics and Univariate Model Estimate for Outcomes Across Parental ACE Categories

Outcome	Parental ACE risk category			Estimate or OR (95% CI)^a^			
	Low risk (n = 32)	Intermediate risk (n = 33)	High risk (n = 14)	Low vs intermediate risk	*P* value	Low vs high risk	*P* value
No. of ED visits and admissions, mean (SD)	3.84 (5.02)	6.33 (6.23)	12.5 (12.8)	Estimate: 2.49 (−1.16 to 6.14)	.18	Estimate: 8.66 (3.94 to 13.38)	<.001
No. of missed clinic visits, mean (SD)	2.97 (4.93)	3.24 (4.34)	5.64 (4.13)	Estimate: 0.27 (−1.98 to 2.53)	.81	Estimate: 2.67 (−0.23 to 5.58)	.07
No. of transfusions, mean (SD)^b^	3.29 (14.3)	1.94 (4.51)	1.29 (3.12)	Estimate: −1.35 (−6.15 to 3.45)	.58	Estimate: −2.00 (−8.18 to 4.17)	.52
Hydroxyurea adherence^c^							
Adherent, No. (%)	11 (34.4)	9 (27.3)	3 (21.4)	OR: 0.98 (0.19 to 0.98)	.98	OR: 4.40 (0.82 to 28.71)	.10
Not adherent, No. (%)	5 (15.6)	4 (12.1)	6 (42.9)				
Penicillin adherence^d^							
Adherent, No. (%)	5 (15.6)	5 (15.2)	4 (28.6)	OR: 0.60 (0.12 to 3.00)	.53	OR: 0.38 (0.05 to 2.33)	.30
Not adherent, No. (%)	10 (31.3)	6 (18.2)	3 (21.4)				

Abbreviations: ACE, adverse childhood experience; ED, emergency department; OR, odds ratio.

^a^ Simple regression analyses. Estimates are given for No. of ED visits and admissions, missed clinic visits, and transfusions. ORs are given for all adherence data.

^b^ Data missing for 1 participant (3.1%) in the low-risk group.

^c^ Data missing for 16 participants (50.0%) in the low-risk group, 20 (60.6%) in the intermediate-risk group, and 5 (35.7%) in the high-risk group.

^d^ Data missing for 17 participants (53.1%) in the low-risk group, 22 (66.7%) in the intermediate-risk group, and 7 (50.0%) in the high-risk group.

**Figure 2.**
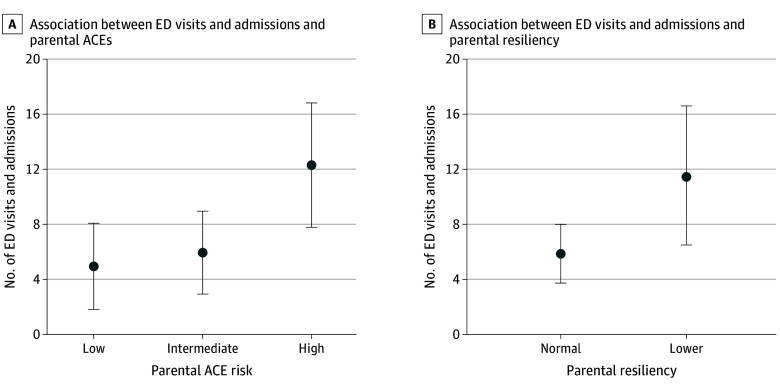
Associations Between Emergency Department (ED) Visits and Admissions and Parental Adverse Childhood Experiences (ACEs) and Resiliency The means and 95% CIs (error bars) were derived from a mixed linear model that included main effects for parental ACEs, parental resiliency, and patient Area Deprivation Index percentile rank scores.

The association between parental ACEs and ED visits and admissions remained significant in the multivariable model that included resiliency and ADI (eTable 2 in Supplement 1). Patients with caregivers in the high-risk ACE category had significantly more ED visits and admissions compared with patients whose caregivers were in the low-risk category (regression coefficient = 7.35; 95% CI, 1.77-12.94) (Figure 2). Furthermore, lower caregiver resiliency was associated with more ED visits and admissions (regression coefficient = 5.69; 95% CI, 0.13-11.26) (Figure 2). By contrast, ADI was not significantly associated with ED visits and admissions (regression coefficient = −0.02; 95% CI, −0.12 to 0.08). Patients of caregivers with high ACEs and low resiliency did not experience a significantly higher number of ED visits and admissions compared with patients whose caregivers had low ACEs and high resiliency (regression coefficient = 11.94, 95% CI, −2.85 to 26.72) (eFigure in Supplement 1); the interaction between parental ACEs and resiliency was not significantly associated with ED visits and admissions (eTable 3 in Supplement 1).

Exploration of the impact of patient diagnosis (severe vs mild) and who completed the ACE-Q (mother vs other) indicated that these variables were not significantly associated with ED visits and admissions (eTable 4 in Supplement 1). Furthermore, excluding data from parents with multiple children did not change the results substantially (eTable 5 in Supplement 1). Parental ACE was not significantly associated with the number of missed clinic visits when parental resiliency and patient ADI were added to the model (eTable 6 in Supplement 1).

## Discussion

Research on the impact of parental ACEs on pediatric health care use is increasing in the primary pediatric care setting^26,41,42^; however, similar work in pediatric patients with high medical complexity is still lagging.^25^ The current study evaluated the association of parental ACEs with health care use in children with SCD. Consistent with our hypothesis and research in other patient populations,^27^ parental ACEs were associated with increased ED use in pediatric patients with SCD. Lower parental resiliency was also independently associated with increased number of ED visits and admissions, although we did not detect a moderating effect of resiliency on the association between ED use and parental ACEs. Health care use is shaped by various factors, notably systematic barriers that limit access to effective care for marginalized populations.^43^ Our study offers insights into parental psychosocial factors that practitioners should consider when caring for pediatric populations from vulnerable communities.^44^

There is robust evidence that ACEs undermine an individual’s health and well-being throughout the life span,^45^ which in turn has sparked interest in parental ACEs.^46,47^ Within the context of ACEs and health care use, some have suggested that parental ACEs present a risk factor for increased stress^27,48,49^ that could interfere with the caregiver’s ability to effectively use health care for their children.^26^ Caregivers of children with chronic illness face unique stressors^50,51^ and experience poorer mental and physical health compared with caregivers with unaffected children.^52^ Moreover, psychosocial disparities are known to exacerbate challenges for caregivers of children with complex health care needs,^51^ a notion that is particularly pertinent in pediatric SCD.^53^ Not only does SCD create a debilitating health burden, it also primarily affects a community that is at high risk of experiencing adversity. Parental ACE was an independent risk factor for increased ED use, even after accounting for factors that are known to affect health care use, such as economic disadvantage.^54,55^

Patients whose caregivers had lower resiliency experienced more ED visits and admissions compared with patients whose caregivers were resilient. However, parental resiliency did not moderate the association between parental ACEs and ED visits. The relatively small number of individuals in the high-risk ACE group likely limited our ability to detect a significant interaction effect. Although caution is warranted in interpreting the resiliency results, it is important to recognize that resilience can be cultivated and practiced.^37,56,57^ Interventions can have a positive effect on patients with chronic illness,^58^ and randomized clinical trials are currently evaluating the effectiveness of resilience-building interventions for individuals with a history of ACEs.^59,60^ Future studies should identify the impact of coping interventions for high-risk populations with chronic illness.

We used ADI as a proxy for sociodemographic conditions of patients’ neighborhoods. Our sample’s ADI national percentile rank scores indicated high levels of deprivation, regardless of parental ACEs. Socioeconomic adversity is common in the state of Arkansas,^61^ where this study was performed. At an estimated $4.3 million, Arkansas has the highest lifetime ACE-related economic burden per adult.^62^ In the current study, there were few observations in the lower, affluent range of the index, which may have limited our ability to detect an association between ACEs and economic deprivation. Critically, risk of mortality increases with increasing ADI scores.^63^ A recent study^64^ found that greater economic deprivation was associated with older brain age. It will be important to continue to examine how sociodemographic adversity affects health and health care use.

### Limitations

The results of this study should be interpreted within the context of its limitations. First, the sample size was relatively small, and the models explained a modest amount of the variance, implying that health care use is affected by a host of factors that were not included in the current study.^65^ Notably, we did not measure chronic pain, which is known to be associated with ACEs and emergency care.^66,67^ Second, families included in the study were approached in the clinic, increasing the potential for sampling bias. Patients who do not regularly attend clinic appointments may use the ED and inpatient services more often; therefore, we may not have fully captured the extent of parental ACEs on pediatric health care use. Relatedly, we were unable to determine the ratio of scheduled clinic visits to missed visits, which would potentially have provided a more accurate picture of health care use. This was a limitation of our institution’s electronic medical record. Third, another limitation of the study is related to the adherence data. Due in part to our data extraction method, which relied on reporting practices of affiliated pharmacies, there was a substantial amount of missing data, and the accuracy of the information could not be verified. Additionally, we did not collect details from the caregiver samples to limit potential linkage between the identity of the respondents and the sensitive survey data. These observations underscore the notion that more prospective research is needed to ensure that all families can interface with the health care system effectively.

## Conclusions

Limitations notwithstanding, our results signal the importance of considering parental ACEs when caring for pediatric patients. The American Academy of Pediatrics published a policy statement in 2021 recommending trauma-informed care^68^ and focusing on developing strategies to identify children who are at high risk for trauma.^22^ Parental ACEs are associated with poor overall health outcomes^23^ and developmental delays in children^41^ and increase a child’s risk to experience ACEs themselves.^69^ Although the importance of parental ACEs in a pediatric health care setting is increasingly recognized, psychosocial services are still primarily focused on the child only rather than the whole family unit.^51^ At a minimum, integrating parental ACE screening into pediatric care could enhance trauma-informed approaches, particularly for high-risk groups such as children with SCD.

## Notes

Supplement 1. **eTable 1.** Distribution of Parental ACEs Among Six Parents With >1 Child **eTable 2.** Model Estimates for Parental ACEs Predicting Number of ED Visits/Admissions **eTable 3.** Model Estimates for Number of ED Visits/Admissions for Mixed Model That Included an Interaction Term for Parental ACEs and Resiliency **eTable 4.** Model Estimates for Number of ED Visits/Admissions for Mixed Model That Explored the Impact of Patient Genotype and Which Parent Completed ACE-Q **eTable 5.** Model Estimates for Multivariable Linear Model for Number of ED Visits/Admissions Without Parents With Multiple Children **eTable 6.** Model Estimates for Parental ACEs Predicting Number of Missed Clinic Visits **eFigure.** Number of ED Visits/Admissions in Pediatric SCD Patients Across Parental ACE Bin (x-axis) and Parental Resiliency (red = resilient; blue = not resilient)

Supplement 2.
Data Sharing Statement
