# Supplementary material for: Parental Adverse Childhood Experiences and Health Care Use Among Children With Sickle Cell Disease
Source: JAMA Netw Open. 2025 Jul 10;8(7):e2519793. doi: 10.1001/jamanetworkopen.2025.19793 (PMC12246875; doi:10.1001/jamanetworkopen.2025.19793)
Supplement: Supplement 1. — eTable 1. Distribution of Parental ACEs Among Six Parents With >1 Child eTable 2. Model Estimates for Parental ACEs Predicting Number of ED Visits/Admissions eTable 3. Model Estimates for Number of ED Visits/Admissions for Mixed Model That Included an Interaction Term for Parental ACEs and Resiliency eTable 4. Model Estimates for Number of ED Visits/Admissions for Mixed Model That Explored the Impact of Patient Genotype and Which Parent Completed ACE-Q eTable 5. Model Estimates for Multivariable Linear Model for Number of ED Visits/Admissions Without Parents With Multiple Children eTable 6. Model Estimates for Parental ACEs Predicting Number of Missed Clinic Visits eFigure. Number of ED Visits/Admissions in Pediatric SCD Patients Across Parental ACE Bin (x-axis) and Parental Resiliency (red = resilient; blue = not resilient) [file jamanetwopen-e2519793-s001.pdf]

Supplemental Online Content

Wilson DK, Crary S, Hobbs G, et al. Parental adverse childhood experiences and health care use among children with sickle cell disease. *JAMA Netw Open*. 2025;8(7):e2519793. doi:10.1001/jamanetworkopen.2025.19793

eTable 1. Distribution of Parental ACEs Among Six Parents With >1 Child
eTable 2. Model Estimates for Parental ACEs Predicting Number of ED Visits/Admissions
eTable 3. Model Estimates for Number of ED Visits/Admissions for Mixed Model That Included an Interaction Term for Parental ACEs and Resiliency
eTable 4. Model Estimates for Number of ED Visits/Admissions for Mixed Model That Explored the Impact of Patient Genotype and Which Parent Completed ACE-Q
eTable 5. Model Estimates for Multivariable Linear Model for Number of ED Visits/Admissions Without Parents With Multiple Children
eTable 6. Model Estimates for Parental ACEs Predicting Number of Missed Clinic Visits
eFigure. Number of ED Visits/Admissions in Pediatric SCD Patients Across Parental ACE Bin (x-axis) and Parental Resiliency (red = resilient; blue = not resilient)

This supplemental material has been provided by the authors to give readers additional information about their work.

© 2025 Wilson DK et al. *JAMA Network Open.*

eTable 1. Distribution of parental ACEs among six parents with >1 child

Parent ID	*Parental ACE*	*Patient ID*
p36	Low Risk	S43
		S44
p71	Low Risk	S95
		S96
p39	Intermediate Risk	S47
		S90
		S91
p51	Intermediate Risk	S62
		S63
p8	Intermediate Risk	S10
		S11
p64	High Risk	S84
		S85

© 2025 Wilson DK et al. *JAMA Network Open.*

eTable 2. Model estimates for parental ACEs predicting number of ED visits/admissions

	**Number of ED visits/admissions**		
Predictors	*Estimates*	*CI*	*p*
(Intercept)	5.28	-2.50–13.06	0.180
ACE Category: Intermediate Risk	1.00	-3.38–5.37	0.651
ACE Category: High Risk	7.35	1.77–12.94	**0.011**
Parental Resiliency Category: Lower Resiliency	5.69	0.13–11.26	**0.045**
Patient ADI Percentile	-0.02	-0.12 – 0.08	0.746
**Random Effects**			
σ^2	26.64		
$\tau_{00\ \text{Parent}}$	33.05		
ICC	0.55		
N_{Parent}	63		
Observations	68		
Marginal R^2 / Conditional R^2	0.203 / 0.644		

© 2025 Wilson DK et al. *JAMA Network Open.*

eTable 3. Model estimates for number of ED visits/admissions for mixed model that included an interaction term for parental ACEs and resiliency

	**Number of ED visits/admissions**		
Predictors	*Estimates*	*CI*	*p*
(Intercept)	5.08	-2.70–12.86	0.196
ACE Category: Intermediate Risk	0.32	-4.32–4.97	0.889
ACE Category: High Risk	5.10	-1.27–11.47	0.115
Parental Resiliency Category: Lower Resiliency	-1.57	-13.18–10.05	0.788
Patient ADI Percentile	-0.01	-0.11–0.09	0.914
Intermediate Risk: Lower Resiliency	7.15	-7.02–21.33	0.317
High Risk: Lower Resiliency	11.94	-2.85–26.72	0.112
**Random Effects**			
σ^2	24.79		
$\tau_{00\ Parent}$	34.60		
ICC	0.58		
N_{Parent}	63		
Observations	68		
Marginal R^2 / Conditional R^2	0.236 / 0.681		

© 2025 Wilson DK et al. *JAMA Network Open.*

eTable 4. Model estimates for number of ED visits/admissions for mixed model that explored the impact of patient genotype and which parent completed ACE-Q

	**Number of ED visits/admissions**		
Predictors	*Estimates*	*CI*	*p*
(Intercept)	4.12	-5.37 – 13.62	0.389
ACE Category: Intermediate Risk	1.20	-3.25 – 5.64	0.593
ACE Category: High Risk	7.58	1.90 – 13.26	**0.010**
Parental Resiliency Category: Lower Resiliency	5.40	-0.27 – 11.07	0.062
Patient ADI Percentile	-0.02	-0.13 – 0.09	0.723
SDC Genotype: Severe	1.92	-2.41 – 6.25	0.378
Who Completed ACE-Q: Other	-0.21	-6.40 – 5.98	0.946
**Random Effects**			
σ^2	27.18		
$\tau_{00\ Parent}$	33.65		
ICC	0.55		
N_{Parent}	63		
Observations	68		
Marginal R^2 / Conditional R^2	0.209 / 0.647		

© 2025 Wilson DK et al. *JAMA Network Open.*

eTable 5. Model estimates for multivariable linear model for number of ED visits/admissions without parents with multiple children

	Number of ED visits/admissions		
Predictors	*Estimates*	*CI*	*p*
(Intercept)	5.63	-1.95 – 13.21	0.142
ACE Category: Intermediate Risk	0.82	-3.48 – 5.12	0.704
ACE Category: High Risk	7.81	2.36 – 13.27	**0.006**
Parental Resiliency: Lower Resiliency	6.46	1.00 – 11.92	**0.021**
Patient ADI Percentile	-0.02	-0.12 – 0.08	0.653

© 2025 Wilson DK et al. *JAMA Network Open.*

eTable 6. Model estimates for parental ACEs predicting number of missed clinic visits

	**Number of Missed Clinic Visits**		
Predictors	*Estimates*	*CI*	*p*
(Intercept)	1.18	-3.73–6.10	0.632
ACE Category: Intermediate Risk	0.18	-2.59–2.96	0.896
ACE Category: High Risk	1.67	-1.86–5.21	0.348
Parental Resiliency Category: Lower Resiliency	2.46	-1.07–6.00	0.168
Patient ADI Percentile	0.02	-0.04–0.09	0.474
**Random Effects**			
σ^2	5.37		
$\tau_{00\ Parent}$	18.35		
ICC	0.77		
N_{Parent}	63		
Observations	68		
Marginal R^2 / Conditional R^2	0.077 / 0.791		

© 2025 Wilson DK et al. *JAMA Network Open.*

eFigure. Number of ED visits/admissions in pediatric SCD patients across parental ACE bin (x-axis) and parental resiliency (red=resilient; blue=not resilient)

The means and 95% confidence limits were derived from a mixed model that included an interaction term or parental ACEs and parental resiliency, as well as a main effect of patient ADI percentile rank scores.

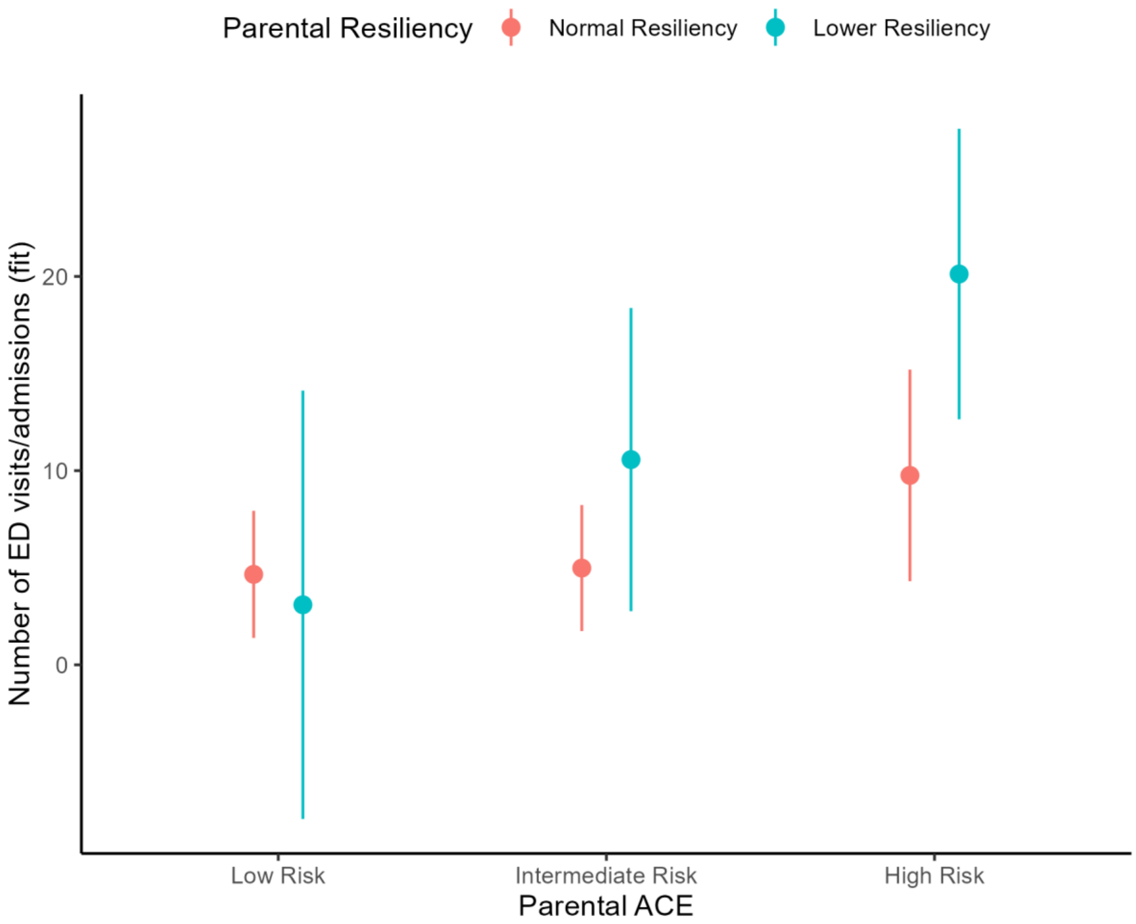

© 2025 Wilson DK et al. *JAMA Network Open.*
