# Supplementary material for: Parental Adverse Childhood Experiences and Health Care Use Among Children With Sickle Cell Disease
Source: JAMA Netw Open. 2025 Jul 10;8(7):e2519793. doi: 10.1001/jamanetworkopen.2025.19793 (PMC12246875; doi:10.1001/jamanetworkopen.2025.19793)
Supplement: Supplement 2. — Data Sharing Statement [file jamanetwopen-e2519793-s002.pdf]

Data Sharing Statement

Wilson. Parental Adverse Childhood Experiences and Health Care Use Among Children With Sickle Cell Disease. *JAMA Netw Open*. Published July 10, 2025.
doi:10.1001/jamanetworkopen.2025.19793

Data
Data available: Yes
Data types: Deidentified participant data
How to access data: evanderplas@uams.edu
When available: With publication

Supporting Documents
Document types: Statistical/analytic code
How to access documents: evanderplas@uams.edu
When available: With publication

Additional Information
Who can access the data: Researchers whose proposed use of the data has been approved
Types of analyses: Any purpose, following institutional data agreement policies.
Mechanisms of data availability: Signed data access agreement
